# Imaging indications and findings in evaluation of lung transplant graft dysfunction and rejection

**DOI:** 10.1186/s13244-019-0822-7

**Published:** 2020-01-03

**Authors:** Mnahi Bin Saeedan, Sanjay Mukhopadhyay, C. Randall Lane, Rahul D. Renapurkar

**Affiliations:** 1 Sections of Thoracic and Cardiovascular Imaging Laboratory, Imaging Institute, Cleveland Clinic, L-10, 9500 Euclid Avenue, Cleveland, OH 44195 USA; 2 Department of Pathology, Cleveland Clinic, Cleveland, USA; 3 Department of Pulmonary and Critical Care Medicine, Cleveland Clinic, Cleveland, USA

**Keywords:** Lung transplantation complications, Primary graft dysfunction, Acute rejection, Chronic lung allograft dysfunction, Imaging findings

## Abstract

Lung transplantation is a treatment option in end-stage lung disease. Complications can develop along a continuum in the immediate or longer post-transplant period, including surgical and technical complications, primary graft dysfunction, rejection, infections, post-transplant lymphoproliferative disorder, and recurrence of the primary disease. These complications have overlapping clinical and imaging features and often co-exist. Time of onset after transplant is helpful in narrowing the differential diagnosis. In the early post transplantation period, imaging findings are non-specific and need to be interpreted in the context of the clinical picture and other investigations. In contrast, imaging plays a key role in diagnosing and monitoring patients with chronic lung allograft dysfunction. The goal of this article is to review primary graft dysfunction, acute rejection, and chronic rejection with emphasis on the role of imaging, pathology findings, and differential diagnosis.

## Key points

- Primary graft dysfunction is a syndrome of acute lung injury after lung transplant, which is characterized by development of hypoxia and diffuse pulmonary opacities in the early post-operative period without another identifiable etiology.
- Acute cellular rejection is the most common type of acute rejection. It is frequently asymptomatic, may occur at any time after transplant, and shows non-specific imaging findings which can vary considerably. Transbronchial biopsy remains the gold standard for diagnosis.
- Antibody-mediated rejection is less common type of acute rejection, and the diagnosis is made by integrating clinical, serologic, radiographic, and pathologic findings.
- Bronchiolitis obliterans syndrome is the most common form of chronic lung allograft dysfunction and characterized by fibrosis/scarring that obliterates bronchioles. It typically develops after the 1st year following transplantation. Bronchiolitis obliterans syndromeis a clinical diagnosis defined by a persistent decline in FEV1 compared with baseline. Bronchiolitis obliterans manifests on CT with bronchial wall thickening, mosaic attenuation, and air trapping on expiratory images.
- Restrictive allograft syndrome is a less common form of chronic lung allograft dysfunction. It carries a worse prognosis and is thought to be characterized pathologically by pleuroparenchymal fibroelastosis. Imaging reveals upper lobe fibrosis and signs of volume loss.

## Introduction

Over the last few decades, lung transplantation has emerged as an accepted treatment option in end-stage lung disease. Diseases such as idiopathic pulmonary fibrosis, advanced emphysema, cystic fibrosis, and pulmonary arterial hypertension are being increasingly treated with lung transplantation. The number of lung transplants has been increasing over the last 20 years, with approximately 64,803 adult lung transplants performed worldwide so far. Improved survival following lung transplant has been linked to multiple factors, including improved surgical technique, drugs, and improved patient selection [1]. Despite the improved outcomes, several complications can develop during the early postoperative period as well as later in the time course, affecting quality of life and contributing to mortality. Important complications in the early postoperative course include surgical complications, infection, primary graft dysfunction, acute rejection, and in later course, chronic lung allograft dysfunction, infection, and post-transplant lymphoproliferative disorder [2, 3]. Difficulties in diagnosis arise from the non-specific clinical presentation of these entities, and the frequent coexistence of multiple complications in the same patient. Imaging plays an important role in evaluating graft dysfunction. Chest radiographs are routinely performed in the postoperative period and for periodic surveillance. Computed tomography (CT) is the imaging test of choice for evaluating lung graft complications and dysfunction. However, because CT findings can be non-specific, especially in the early postoperative period, knowledge of the clinical context, including symptoms, time course of the presentation since transplant, and correlation with other investigations such as pulmonary function tests, echocardiography, and bronchoscopy helps in narrowing the differential diagnosis (Table 1) [2–4]. Other imaging tools such as magnetic resonance imaging (MRI) and positron-emission tomography (PET) are helpful in specific clinical scenarios.

**Table 1:** Summary of role of additional common tests in the workup of graft dysfunction

Workup	Uses
Bronchoscopy	BAL: to exclude infection. Infection is considered in the differential of PGD, acute rejection (ACR, AMR), and BO/BOS.
	TBB:
	ACR: histopathological findings are the gold standard diagnostic test.
	AMR: histopathological findings and positive C4d stain are suggestive diagnostic features.
	BO: pathological findings are characteristic; disease is patchy and biopsy may be negative; not required for diagnosis.
	Recurrence of the primary lung disease such as sarcoidosis.
	Malignancy such as post-transplant lymphoproliferative disorder.
	Airway:
	Dehiscence in early post-operative period.
	Stenosis and malacia can cause abnormal spirometry; differential diagnosis of BOS.
Spirometry	Sensitive in detecting graft dysfunction;
	BOS: irreversible obstructive pattern; > > 20% decline in FEV_1_ of baseline
	RAS: irreversible restrictive pattern; > 20% decline in FEV_1_ of baseline.
Echocardiography	Cardiac dysfunction: cardiogenic edema or volume overload may cause diffuse lung opacities and should be considered in the differential of PGD in particular.
CTA	Pulmonary embolism
	Pulmonary venous thrombosis: in the differential diagnosis of PGD.
GERD Workup	Aspiration: in the differential of PGD and acute rejection; may co-exist with either one; it is a potential risk factor of BO/BOS

*BAL*, bronchoalveolar lavage; *PGD*, primary graft dysfunction; *ACR*, acute cellular rejection; *AMR*, antibody-mediated rejection; *BOS*, bronchiolitis obliterans syndrome; *TBB*, transbronchial biopsy; *FEV*_*1*_, forced expiratory volume in 1 s; *RAS* restrictive allograft syndrome

The goal of this article is to review the pathophysiology and evaluation of lung transplant graft dysfunction along a time continuum with emphasis on the role of imaging.

### CT protocol

The CT protocol used depends on the clinical question that needs to be addressed. For routine follow-up and evaluation of lung parenchyma, a routine chest CT is sufficient. Use of contrast is optional and preferred if there is a clinical concern of vascular complications. For acute graft dysfunction, CT angiography can be helpful if vascular complications such as pulmonary artery stenosis/occlusion or pulmonary venous stenosis are suspected. For evaluation of chronic lung allograft dysfunction, CT protocol in patients in our institution includes high-resolution CT images from the lung apex to the diaphragm at end-inspiration with 1-mm slice thickness in 1-mm increments. End-inspiration imaging is usually followed by a free breathing imaging at different levels (mid trachea, carina, lung bases). Free breathing imaging is helpful in assessing for air way malacia and air trapping. Axial (3 × 1.5 mm), sagittal (3 × 3 mm), and coronal (3 × 3 mm) images are routinely reconstructed. Continuous axial 1-mm images are available upon request to be uploaded to a 3-dimensional workstation for further evaluation including virtual bronchoscopy if needed but not routinely reconstructed.

## Hyperacute rejection

Hyperacute rejection is a type of antibody-mediated rejection. Hyperacute rejection after lung transplant is exceedingly rare in the era of sensitive pre-transplant panel reactive antibody testing. Hyperacute rejection occurs in patients with pre-formed circulating antibodies to donor human leukocyte antigen [HLA] that attack the graft. It develops during surgery or within the first 24 h after lung transplant. It can be treated with apheresis and augmented immunosuppression, but can be fatal despite treatment. Initial radiographs typically show diffuse opacities in the transplanted lung(s), typically of pulmonary edema pattern [5–9].

## Primary graft dysfunction

**Fig. 1:**
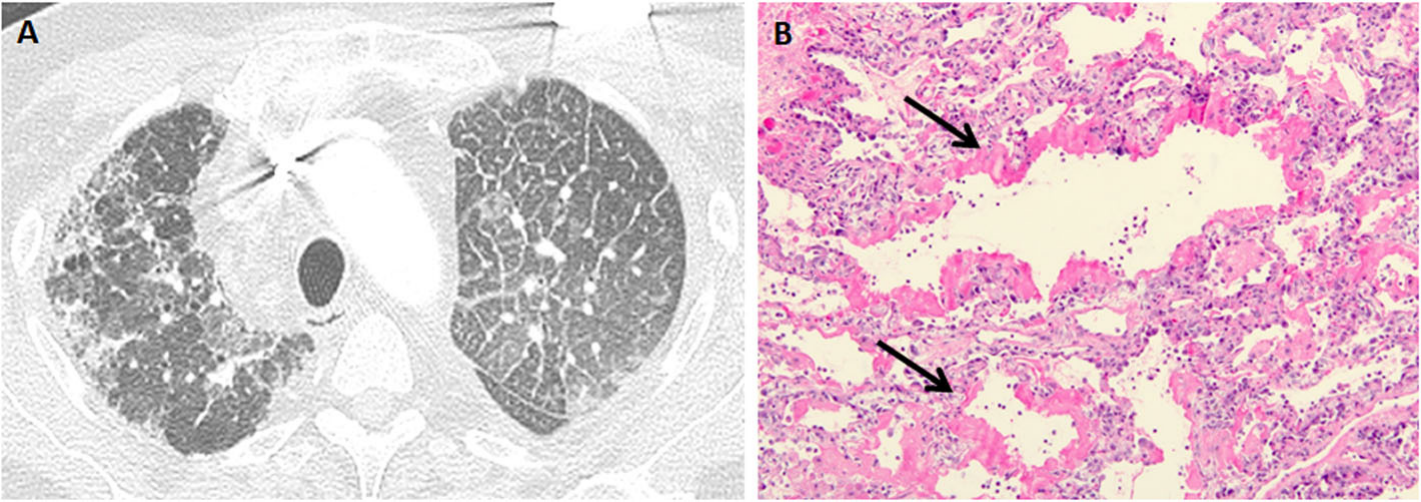
Primary graft dysfunction: imaging and transbronchial biopsy findings. The patient was 2 days status-post left lung transplant and developed increasing hypoxemia. Axial CT images (**a**) shows smooth interlobular septal thickening with ground-glass opacities in the transplanted left lung. These findings are typical of primary graft dysfunction but are indistinguishable from acute rejection. Transbronchial biopsy (**b**) from a different lung transplant patient with primary graft dysfunction showing diffuse alveolar damage. Note hyaline membranes (arrows)

Primary graft dysfunction is a syndrome of acute lung injury in the early post-transplant period. It is a major cause of early morbidity and mortality, with an incidence in the range of 30% [10]. Primary graft dysfunction is thought to result from multifactorial injury to the transplanted lung by the transplant process and other contributing factors. Transplant process-related factors include organ retrieval, preservation, implantation, and reperfusion. Acid aspiration, pneumonia, and micro-trauma from mechanical ventilation are thought to be contributing factors. The term “primary graft dysfunction” has replaced other previously used terms such as ischemia-reperfusion injury/edema, re-implantation edema/response, and primary graft failure. The main pathologic manifestation of primary graft dysfunction is diffuse alveolar damage, characterized by hyaline membranes in the acute stage (Fig. 1) and alveolar septal thickening by fibroblasts [10, 11]. The pathologic findings are identical to those seen in acute interstitial pneumonia, except that they occur in the context of lung transplantation [12]. Survivors of primary graft dysfunction have a higher incidence of development of chronic lung allograft dysfunction [10, 13, 14].

Primary graft dysfunction is characterized by the development of hypoxia and diffuse pulmonary radiographic opacities within the first 72 h after lung transplantation without another identifiable cause such a cardiogenic pulmonary edema, infection, or rejection. Assessment for this syndrome occurs at four time points, starting at the time of reperfusion of the second lung, and then at 24, 48, and 72 h. The goal of the grading system for primary graft dysfunction is to help identify patients with potentially poor outcomes. The ratio of arterial fraction of oxygen (PaO2)/fraction of inspired oxygen (FiO2) determines the severity of primary graft dysfunction. Any PaO2/FiO2 ratio with a normal chest radiograph is considered grade 0. Grades 1, 2, and 3 are characterized by diffuse pulmonary opacities on chest radiograph with PaO2/FiO2 > 300, between 200 and 300, and < 200, respectively [10].

Radiographic and CT findings of primary graft dysfunction are variable and non-specific. The most common findings are perihilar and basilar heterogeneous opacities. The opacities typically appear within 3 days after transplantation, worsen for a few days, and usually start clearing between 5 and 10 days post-transplant. Persistent or worsening opacities should raise concern for acute rejection or infection. Typically, focal clustered opacities raise the suspicion of an infectious process [2, 15–17]. Chest CT may show ground-glass opacities, consolidation, and interstitial and interlobular septal thickening (Fig. 1) [2, 3, 18].

**Fig. 2:**
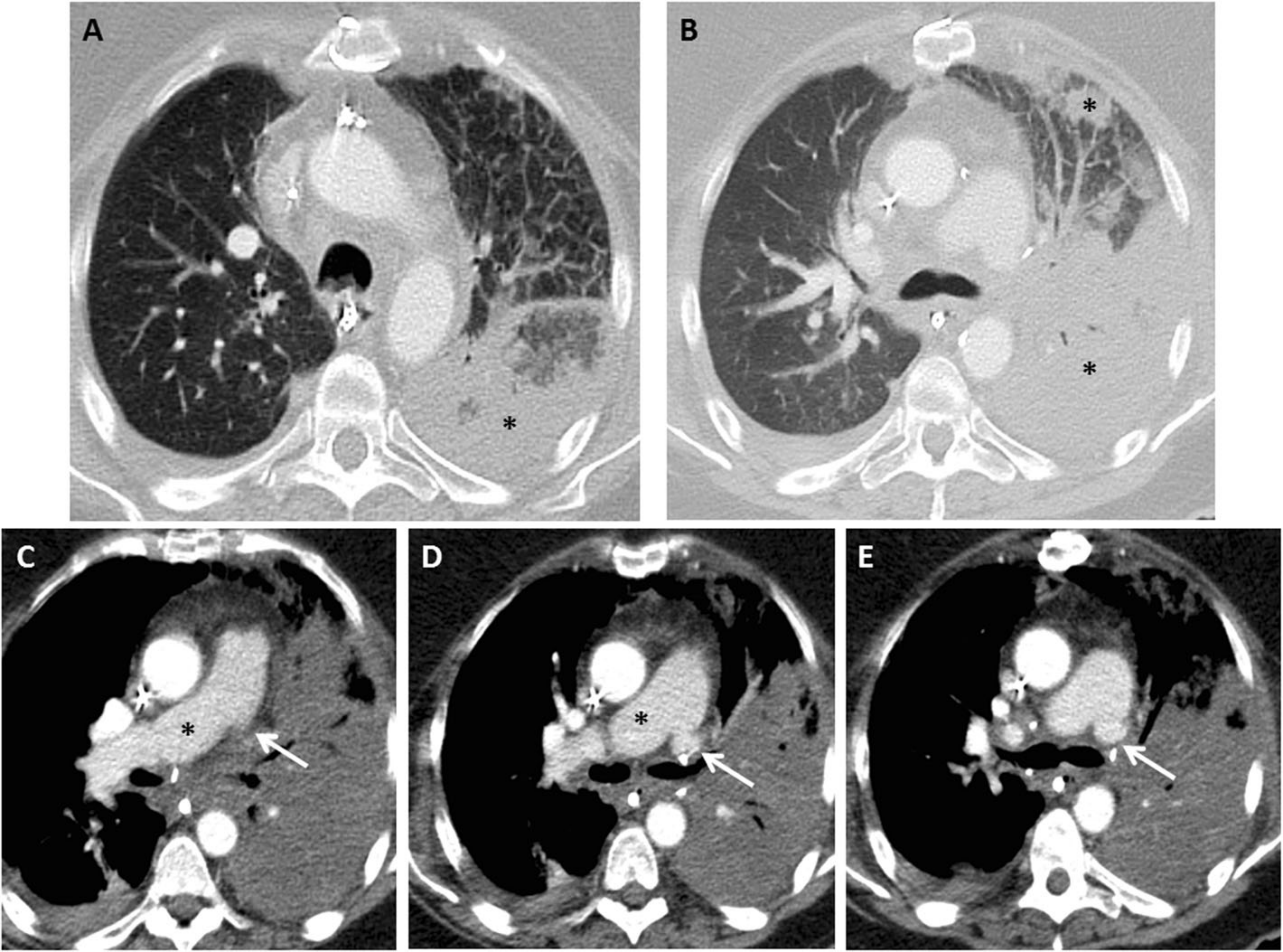
Left pulmonary artery anastomotic occlusion. CT-PE protocol was performed after suspecting left pulmonary artery anastomotic occlusion on initial non-enhanced CT scan performed on day 3 post bilateral lung transplant. Axial images using lung window (**a** and **b**) show diffuse and ill-defined consolidations in the left lung, predominantly in the lingula and the left lower lobe (asterisks). These findings are likely related to areas of pulmonary hemorrhage with evolving areas of pulmonary infarcts. There is associated ground glass opacities and interlobular septal thickening, best appreciated in the left upper lobe. Axial images using soft tissue window (**c**–**e**) show a total occlusion of the left pulmonary artery at the arterial anastomotic site (arrows) and a patent right pulmonary artery (asterisks). Note the enhancing left pulmonary veins within the left lung consolidation

Differential diagnostic considerations for graft dysfunction during the first 72 h include pulmonary edema (due to volume overload or myocardial dysfunction), pneumonia, rejection, aspiration, pulmonary, and pulmonary vein stenosis or airway anastomotic complications. Primary graft dysfunction may coexist with any of these processes. Therefore, the diagnostic workup of suspected primary graft dysfunction should include characterization of radiographic abnormalities, assessment of volume status, echocardiography, review of ventilator settings, review of donor-recipient crossmatch, and screening for donor-specific antibodies [4]. The primary role of imaging is to exclude other process that may cause symptoms or radiographic abnormalities. For example, CT angiography can be used to diagnose pulmonary embolism or arterial or venous anastomotic occlusion or stenosis (Fig. 2) [2]. Imaging also helps in selecting the most appropriate site for bronchoalveolar lavage and transbronchial biopsy [19]. Flexible bronchoscopy with bronchoalveolar lavage is usually performed in cases of early graft dysfunction with infiltrates to obtain samples for microbiologic testing, to inspect the airway, and to evaluate for bronchial anastomotic complications [4].

## Acute allograft rejection

**Fig. 3:**
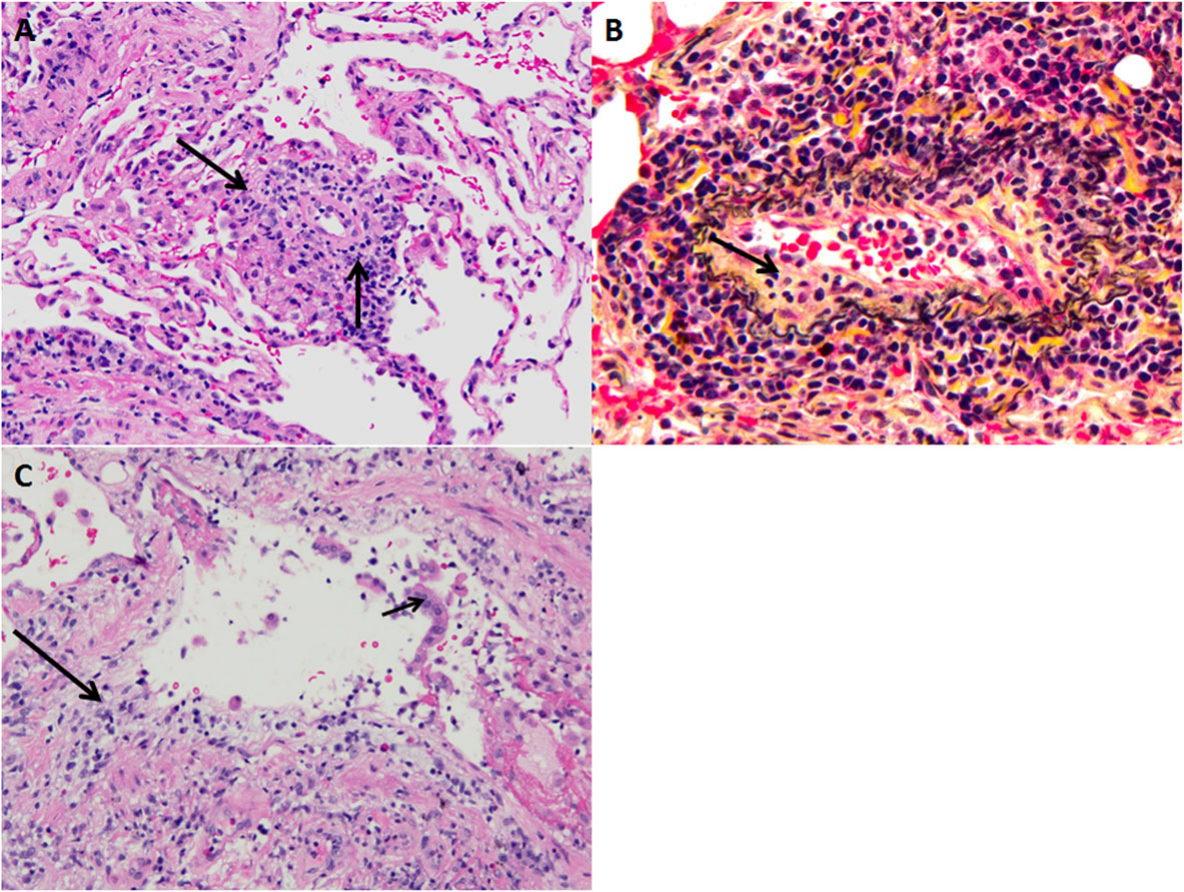
Transbronchial biopsy findings in acute cellular rejection following lung transplantation. The vascular component of acute cellular rejection (**a**) is characterized by a lymphocyte-rich inflammatory infiltrate involving and surrounding small blood vessels within lung parenchyma. The Movat pentachrome stain (**b**) helps to highlight elastic layers in the blood vessel, demonstrating that inflammatory cells breach the elastic layers (black wavy lines) and involve the vascular intima (arrow). Airways involved by acute cellular rejection (**c**) show inflammation (long arrow). In more advanced examples, there is epithelial damage, manifested by sloughing of epithelial cells into the airway lumen (short arrow)

Acute rejection occurs in more than a third of lung transplant cases in the first year after transplant, and results in approximately 4% of deaths within the first 30 days after transplantation [20–22]. Acute cellular rejection is the most common type of acute rejection. It is caused by a T-lymphocyte-rich cellular response directed against major histocompatibility complexes (MHC) in the donor lung (also known as HLA in humans), or other donor antigens. Acute cellular rejection can occur at any time after transplant and is most commonly encountered within the first year after lung transplantation [22]. Acute rejection may present with symptoms of dyspnea, cough, or lower extremity edema. A decline in forced expiratory volume in 1 s (FEV1) may be noted on pulmonary function testing. Frequently, patients will be asymptomatic with stable pulmonary function testing, and the diagnosis is made with pathologic findings in transbronchial biopsies obtained during surveillance bronchoscopy. The pathologic findings are well-characterized [22–24], and include patchy lymphohistiocytic inflammatory infiltrates centered on small blood vessels (arterioles/venules). Bronchioles may also be inflamed. The International Society for Heart and Lung Transplantation (ISHLT) has laid out standardized nomenclature for grading of acute cellular rejection based on the severity of the inflammatory process and the structures involved. The vascular component, which is most common in practice, ranges from grade A0 (no rejection) to grade A4 (severe), most cases being A1 or A2. The airway component ranges from B0 (no rejection) to B2R (high grade). Acute vascular and airway rejection can be seen independently (Fig. 3), or may co-exist in the same patient [23].

**Fig. 4:**
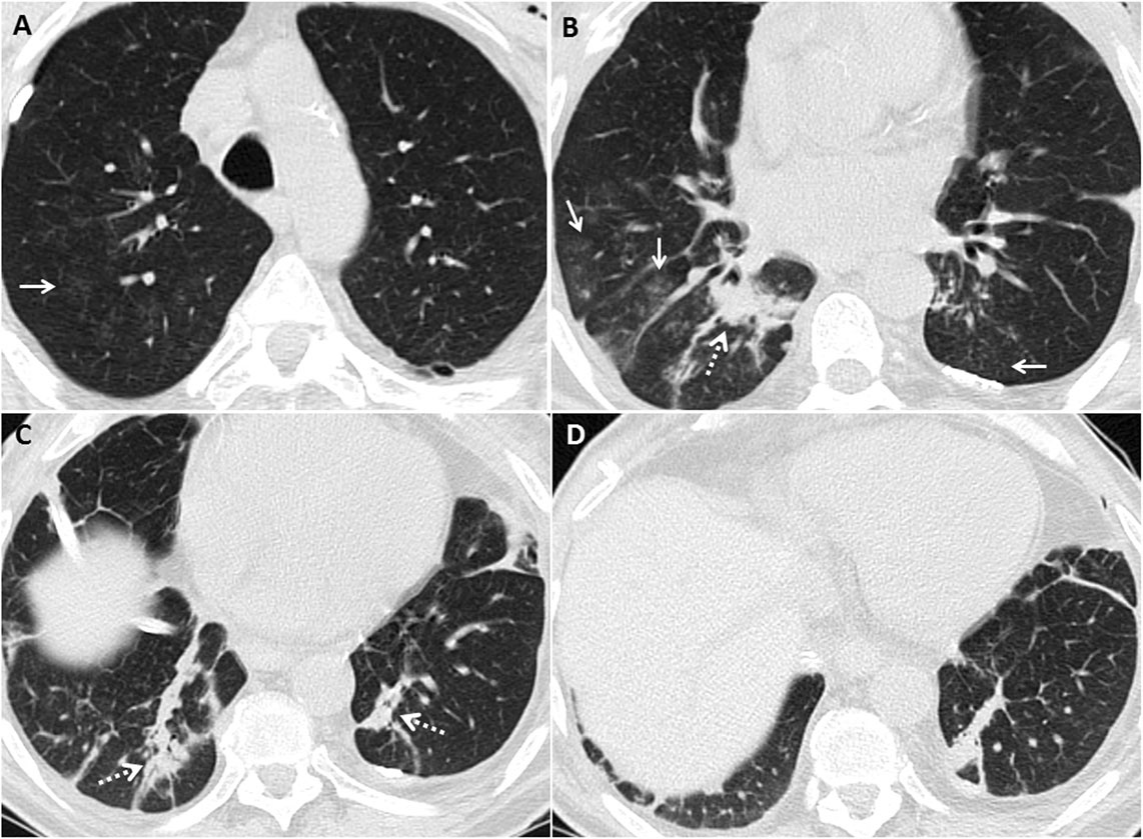
Patient with bilateral lung transplant and mild acute cellular rejection. Axial CT images (**a**–**d**) show asymmetric (right more than left) patchy and nodular peribronchial ground glass opacities (arrows) with basilar peribronchial consolidative opacities (arrowheads) and mild bibasilar septal thickening. Transbronchial biopsy of both lower lobes showed mild acute cellular rejection (grade A2 B0)

Chest radiography is often ordered as a baseline test. It may show non-specific findings such as perihilar opacities and/or pleural effusion. It has low sensitivity and specificity for acute rejection, and is usually performed to look for diseases other than rejection. Chest CT is typically obtained to evaluate the severity and distribution of the disease process. The CT findings of acute cellular rejection are non-specific. They include ground-glass opacities, consolidation, and interstitial thickening with or without pleural effusions (Fig. 4) [3, 25, 26]. Significant clinical and radiographic improvement after early administration of high dose of corticosteroids provides additional supporting evidence for acute rejection [27]. Given the non-specific nature of imaging findings in acute cellular rejection, the main role of CT is to exclude other etiologies and help identifying targets for bronchoscopic tissue sampling [22, 25, 26]. Trans-bronchial biopsies are typically obtained from the lower lobes if the lungs are normal on imaging or if the disease process is diffuse. When disease is patchy on imaging, biopsies are usually taken from the radiologically abnormal areas [19].

**Fig. 5:**
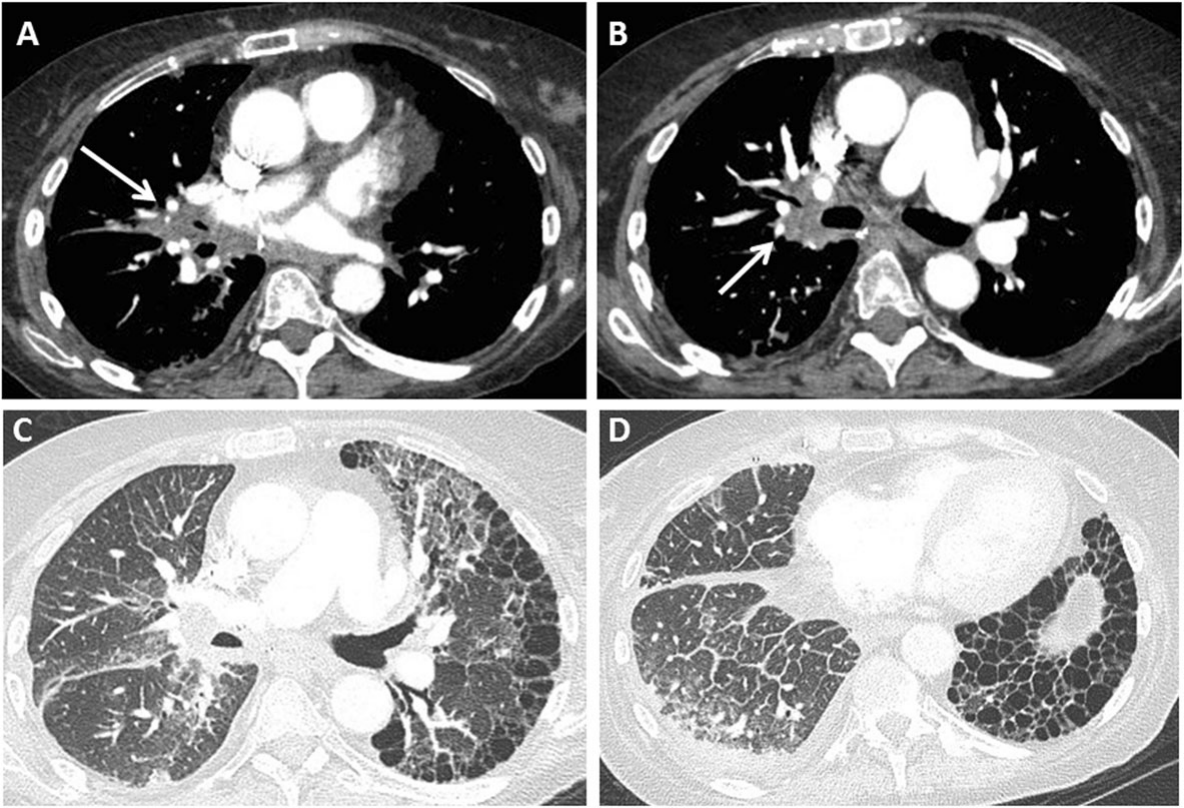
A 60-year-old male with rheumatoid arthritis–related usual interstitial pneumonia status post right lung transplant presented with shortness of breath. CT-PE protocol was performed. Axial images using soft tissue (**a** and **b**) and lung (**c** and **d**) windows show infiltrative right hilar and perihilar soft tissue density (arrows), peribronchovascular interstitial thickening, interlobular septal thickening, and ground-glass opacities within the transplanted right lung. Advanced fibrosis with exuberant honeycombing is noted in the native left lung. These findings raised concern for post-transplant lymphoproliferative disorder. Pathology and EBV PCR were negative. Flow cytometry was positive for donor specific antibody to DQ2. These findings were suggestive of antibody-mediated rejection

Antibody-mediated rejection is less common than acute cellular rejection. It is caused by antibodies against HLA antigens in the donor lung, or possibly to auto-antigens in the context of lung injury. Patients with antibody-mediated rejection usually present with respiratory symptoms that typically manifest weeks or months following transplantation. The diagnosis is extremely challenging and needs careful correlation of the clinical, radiologic, pathologic, and serologic findings (Fig. 5). The pathologic findings in this entity are poorly defined. Reported findings include acute lung injury and diffuse alveolar damage with or without capillaritis. Diagnostic criteria include detection of circulating donor specific antigen (DSA), compatible histopathologic findings, and positive immunohistochemical staining for complement component 4d (C4d). Cases with all three features are diagnosed as “definite.” The recommended designation is “probable” if 2 features are present, and “possible” if any 1 feature is present [5]. In practice, immunohistochemical staining for C4d is associated with several problems, including low sensitivity and specificity, and high background staining. There is limited data in the literature regarding the imaging findings of acute antibody-mediated rejection.

**Fig. 6:**
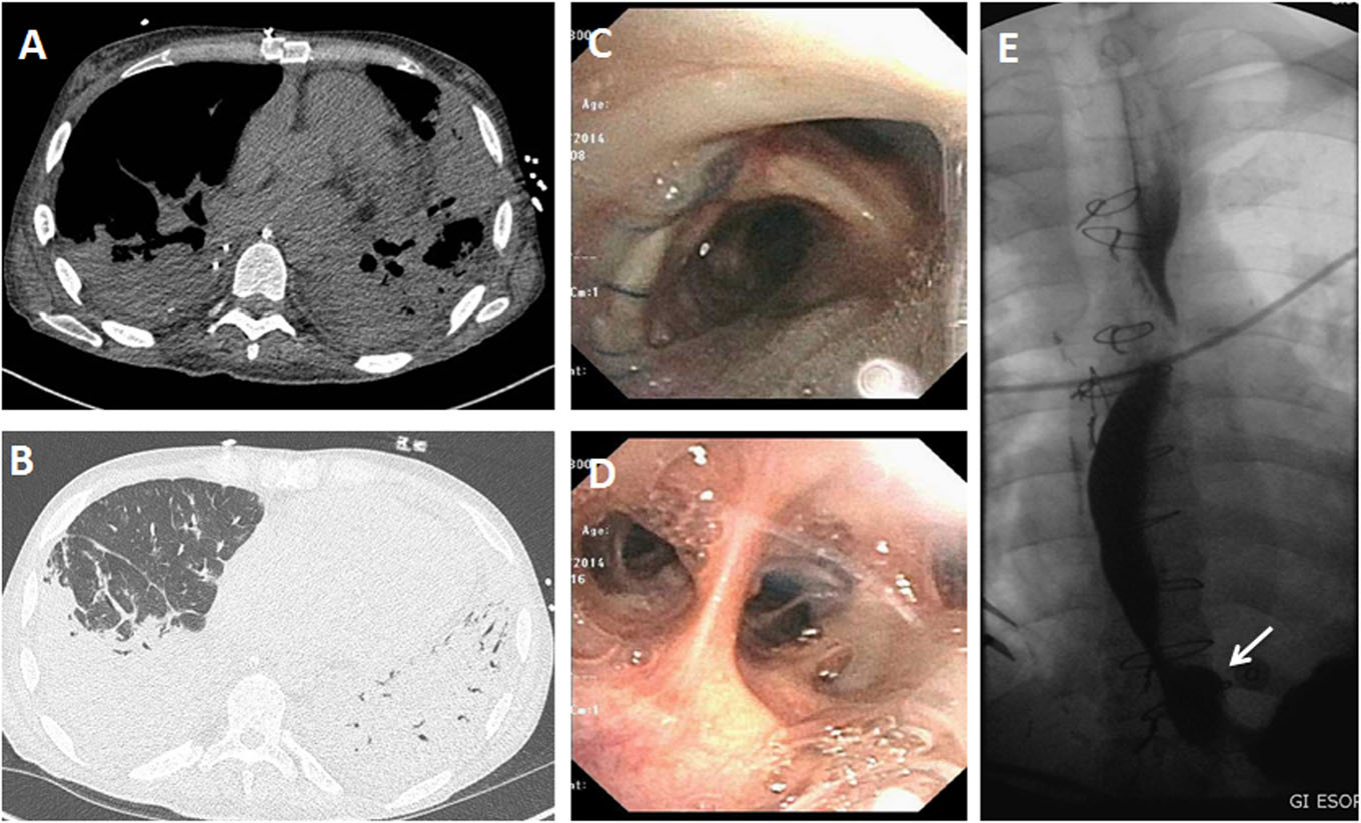
Role of multimodality imaging in a 32-year-old male status post bilateral lung transplant presenting with shortness of breath. CT images in lung window setting (**a**, **b**) show multifocal bilateral consolidations, which are predominately seen in both lower lobes. Images from flexible bronchoscopy (**c**, **d**) show mucopurulent secretions at origin of right bronchus and in right middle lobe bronchi. Still image from esophagogram (**e**) shows small hiatal hernia (arrow); the patient had delayed esophageal emptying. Mycoplasma IgM titers were high and the patient was felt to have mycoplasma pneumonia with co-existing aspiration pneumonia. Trans-bronchial biopsy was negative for acute rejection

The differential diagnosis of lung transplant patients with declining allograft function weeks to months after transplant includes acute rejection, infection, airway complications (airway stenosis, tracheobronchomalacia, or granulation tissue), vascular anastomotic complications, chronic rejection, and cardiac dysfunction [4, 22]. Evaluation includes a battery of laboratory testing for evidence of infection, imaging with chest radiography or CT, spirometry, and flexible bronchoscopy for bronchoalveolar lavage and transbronchial biopsy to confirm the diagnosis of acute rejection and to exclude processes such as infection, aspiration or organizing pneumonia [4, 22]. Even after a thorough evaluation, some cases can prove diagnostically challenging. The evaluation of these patients can be greatly complicated by presence of more than one process in the same patient (Fig. 6). Post-transplant lymphoproliferative disorder could rarely present early during the first weeks post lung transplant and should be considered in the differential diagnosis of persistent opacities during this period [28].

## Chronic lung allograft dysfunction

Chronic lung allograft dysfunction (CLAD) following lung transplantation is a major cause of morbidity and mortality [29]. It is an umbrella term that encompasses bronchiolitis obliterans syndrome and restrictive allograft syndrome [29, 30].

### Bronchiolitis obliterans syndrome/obliterative bronchiolitis

**Fig. 7:**
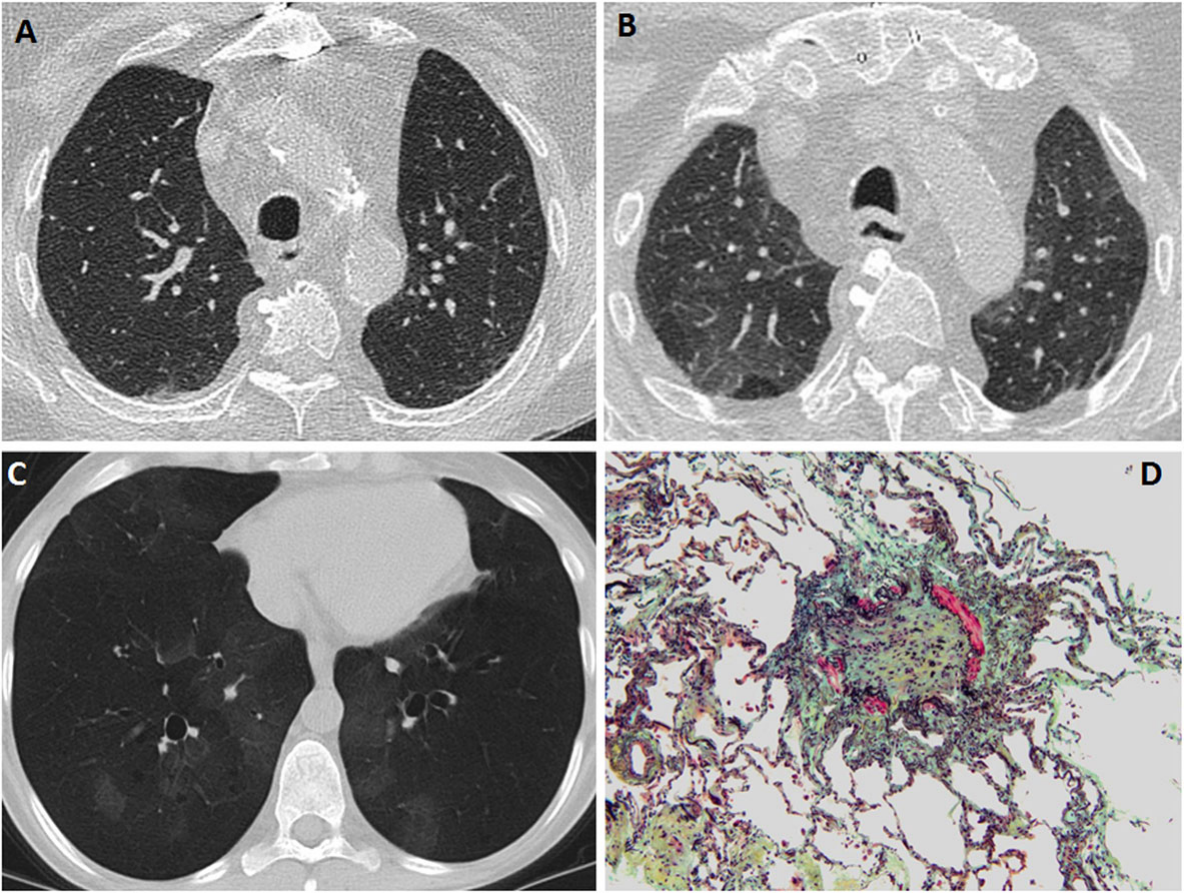
Bronchiolitis obliterans syndrome. Axial inspiratory (**a**) and expiratory (**b**) CT images show normal-appearing transplanted lungs on inspiratory phase and areas of air-trapping on expiratory phase. CT minimum intensity projection (MinIP) image (**c**) of a different patient shows mosaic attenuation and bronchiectasis. Pathologic findings of obliterative bronchiolitis (D); the lumen of this bronchiole is completely obliterated by fibrous tissue (green and yellow). Residual smooth muscle bundles: red. Elastic tissue: black (Movat pentachrome stain, 100×)

Bronchiolitis obliterans syndrome/obliterative bronchiolitis is the most common form of chronic lung allograft dysfunction and reported in 48% of patients by 5 years after transplant and 76% of patients after 10 years [1]. Obliterative bronchiolitis (also known as constrictive bronchiolitis) is a pathologic diagnosis characterized by fibrotic scarring that greatly narrows or completely obliterates bronchiolar lumens (Fig. 7). Bronchiolitis obliterans syndrome is the clinical manifestation of obliterative bronchiolitis. The hallmark of this syndrome is the presence of declining pulmonary function tests compared with baseline, in the absence of another explanation such as acute rejection, infection, pulmonary edema, or airway stenosis. Defined in this way, the diagnosis obviates the need for pathologic confirmation, which is limited by sampling issues and low sensitivity. Obliterative bronchiolitis/bronchiolitis obliterans syndrome is the most common manifestation of chronic lung allograft dysfunction [29]. Acute rejection, recurrent infections, primary graft dysfunction, HLA mismatch, cytomegalovirus pneumonitis, aspiration, and non-compliance with immunosuppressive therapy are thought to be potential risk factors for this disease [31]. Bronchiolitis obliterans syndrome often presents with non-specific symptoms; in advanced cases, patients may present with dyspnea at rest. The key feature is irreversible airflow limitation with a sustained decrease in FEV1 for > 3 weeks. The clinical staging of bronchiolitis obliterans syndrome is based on the FEV1 compared with baseline (Table 2). Pathologic examination shows obliterative bronchiolitis, as described above [29, 32]. Bronchiolar involvement in this disease is typically patchy, greatly limiting the yield of transbronchial biopsy [33]. A major role of bronchoscopy in this setting is to exclude other causes of decreased FEV1 such as acute rejection, infection, or bronchial stenosis [29].

**Table 2:** BOS classification system

	FEV_1_	FEV_25-75_
BOS 0	> 90% of baseline	> 75% of baseline
BOS 0-p	81–90% of baseline	< or = 75% of baseline
BOS 1	66–80% of baseline	
BOS 2	51–60% of baseline	
BOS 3	< 50% of baseline	

*BOS*, bronchiolitis obliterans syndrome; *BOS 0-p*, potential BOS; *FEV*_*1*_, forced expiratory volume in 1 s; *FEV*_*25-75*_, forced expiratory flow at mid-expiratory phase (25–75%)

Unlike assessment of acute rejection, imaging plays a crucial role in the diagnosis and stratification of patients with chronic lung allograft dysfunction. Chest radiography is often the first investigation and is often normal in the early stage. Volume loss with fibrotic changes can be seen in advanced disease. CT scans may be normal during early stages of the process, or may show air trapping on expiratory imaging reflecting early obliterative bronchiolitis [34]. Late stages show mosaic attenuation caused by air trapping and may be associated with bronchiectasis (Fig. 7) [35–38]. Spirometry is more sensitive in detecting the bronchiolitis obliterans syndrome than chest CT [39]. Tree-in-bud and centrilobular nodules can be seen and are thought to represent mucus impaction in distal bronchioles. Respiratory infection should be excluded based on clinical symptoms, bronchoalveolar lavage, and absence of concomitant suggestive imaging features such as consolidation before attributing these findings to the bronchiolitis obliterans syndrome [40].

### Restrictive allograft syndrome

**Fig. 8:**
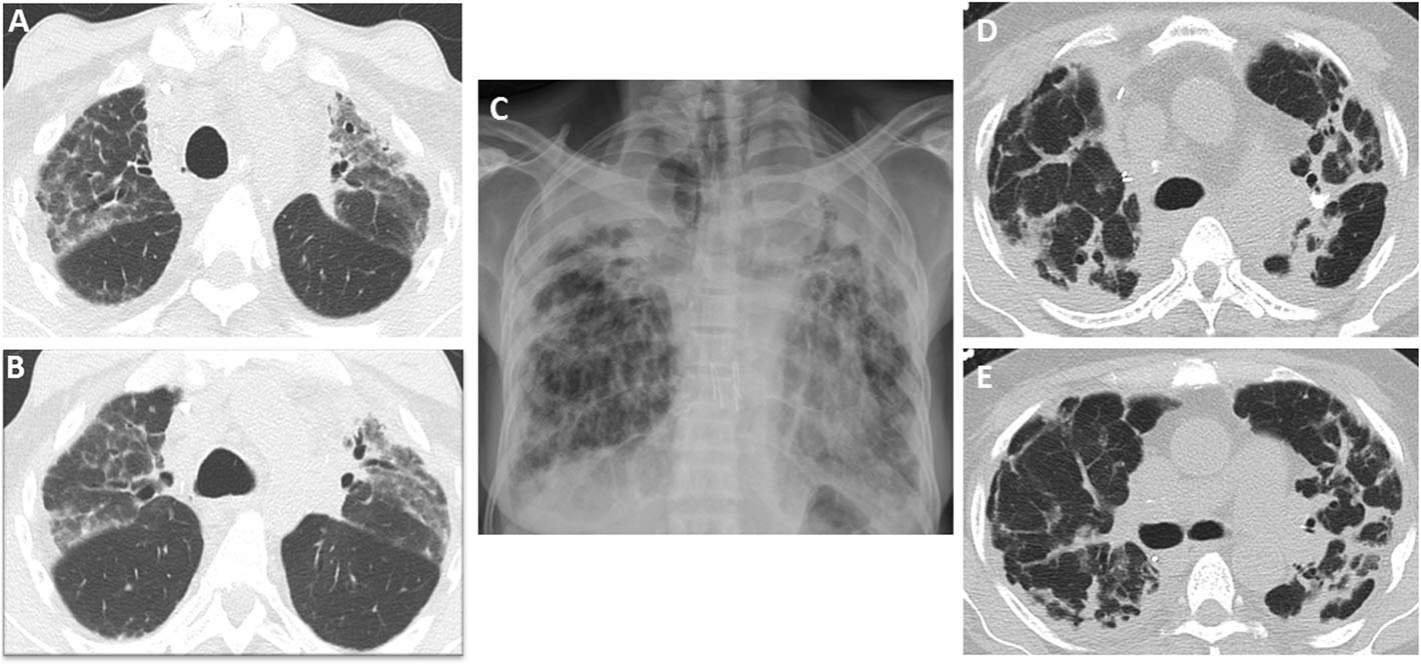
Spectrum of imaging findings in restrictive allograft syndrome. CT images (**a** and **b**) show early to intermediate changes with upper lobe ground glass opacities and mild volume loss. Chest radiograph (**c**) in a patient with advanced restrictive allograft syndrome showing upper lobe predominant subpleural pleuroparenchymal fibrotic changes with volume loss. CT images (**d** and **e**) in another patient with advanced restrictive allograft syndrome showing predominant subpleural fibrotic changes with associated pleural thickening

Restrictive allograft syndrome is the less common phenotype of irreversible chronic lung allograft dysfunction; it has been associated with a worse prognosis compared with bronchiolitis obliterans syndrome. It ultimately affects approximately 10% of lung transplant recipients. Patients present with restrictive lung function tests [30]. Reported pathologic findings include pleuroparenchymal fibroelastosis with or without organizing pneumonia [29, 41]. Early imaging findings include central and peripheral ground-glass opacities and organizing pneumonia (Fig. 8) [42]. Imaging features that suggest organizing pneumonia include peribronchial, perilobular, or subpleural consolidations with or without the classic reverse halo sign [43]. Peripheral fibrotic changes with upper lung predominance, volume loss, hilar retraction, and traction bronchiectasis (Fig. 8) are the classic advanced imaging findings [30, 42, 44]. Some cases may present with lower lobe or diffuse opacities and tend to have poor survival [30].

**Fig. 9:**
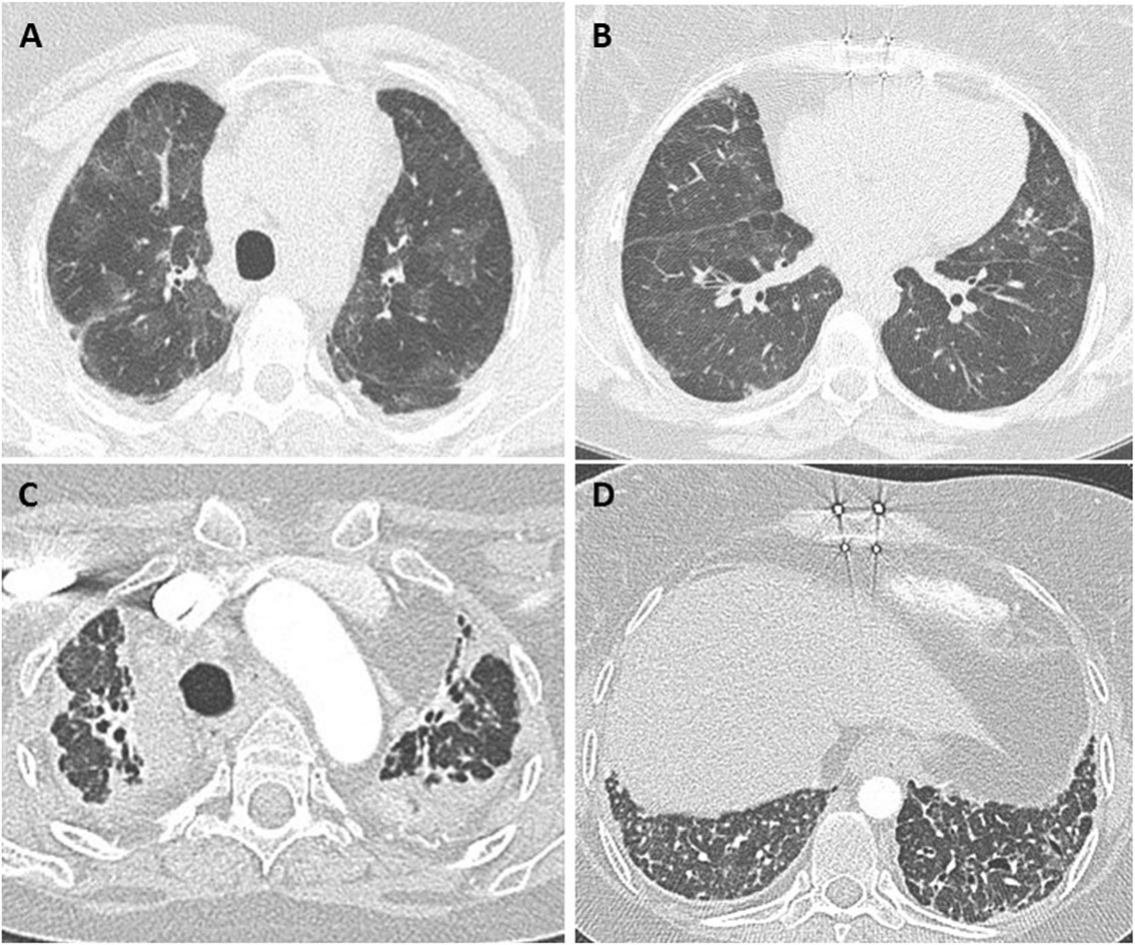
Imaging findings showing evolution of bronchiolitis obliterans to restrictive allograft syndrome. The patient initially presented with decreased FEV1 and mid-flows. Initial axial CT images (**a** and **b**) show mosaic attenuation, likely due to air-trapping. Transbronchial biopsy was negative for infection and acute rejection and the diagnosis of bronchiolitis obliterans syndrome was made. Follow-up CT images (**c** and **d**) after 3 years show evolution of changes with upper lobe predominant subpleural pleuroparenchymal fibrotic changes typical of restrictive allograft syndrome

Survival estimates range from 6 to 18 months, compared with 3–5 years in patients with bronchiolitis obliterans syndrome. It is important to note that chronic lung allograft dysfunction is a heterogeneous umbrella term, and that these phenotypes can coexist, or evolve from one to the other (Fig. 9). Evolution from obstructive to restrictive chronic lung allograft dysfunction is more common [30, 45]. The underlying severe obstruction poses a diagnostic challenge in cases that evolve from bronchiolitis obliterans syndrome to restrictive allograft syndrome. However, these cases carry a better prognosis compared with those without preceding bronchiolitis obliterans syndrome [45].

Additional imaging techniques that may allow early detection and monitoring of chronic lung allograft dysfunction include quantitative CT and low-dose CT volumetry. Quantitative CT is a density mapping with expiratory/inspiratory-ratio mean lung density to detect pathologic air trapping in lung transplant patients [46]. Advanced quantitative analysis of CT in lung transplant patients can improve the accuracy of diagnosis of bronchiolitis obliterans syndrome and in predicting functional parameters such as FEV [47]. Low-dose CT volumetry shows decreased lung volume in restrictive allograft syndrome, whereas patients with bronchiolitis obliterans syndrome show no significant post-transplant change [48]. Oxygen-enhanced T1-mapping MRI might become a noninvasive tool for early detection and monitoring of bronchiolitis obliterans syndrome. Oxygen is used as an inhaled contrast agent with T1 weighted images which may provide information regarding lung ventilation, perfusion, and diffusion capacity. The oxygen transfer function is a parameter using T1 relaxivity measured at room air and at 100% oxygen. Decreasing oxygen-induced relaxivity and increasing heterogeneity are suggestive features of bronchiolitis obliterans syndrome [49]. This technique requires breath hold and use of a closed facemask, which may not be well tolerated by some patients. Therefore, some simplified noncontrast-enhanced lung ventilation techniques have emerged including Fourier decomposition MRI (FD-MRI) and phase resolved functional lung MRI (PREFUL), which may have a potential applications in the future for early detection and severity assessment of chronic lung allograft dysfunction [50, 51]. The uptake of Fluorine-18-Fluorodeoxyglucose (FDG) on PET imaging may be a promising diagnostic and prognostic tool in cases of chronic lung allograft dysfunction. FDG uptake on fused PET/CT scan may distinguish restrictive allograft syndrome from bronchiolitis obliterans syndrome cases. Also, patients with restrictive allograft syndrome and areas of avid FDG uptake tend to have worse outcome [52].

## Conclusion

It is important to know the various types of lung allograft dysfunction along a time continuum (Table 3). Imaging findings in the acute setting (primary graft dysfunction and acute rejection) are non-specific and need to be interpreted in conjunction with the clinical picture and other investigations. In contrast, imaging plays a key role in the diagnosis and follow-up of patients with chronic lung allograft dysfunction. Quantitative CT and T1-mapping MRI might evolve as non-invasive markers for chronic lung allograft dysfunction in the near future.

**Table 3:** Time onset, radiological findings, and diagnostic features of lung transplant primary graft dysfunction and different types of rejection

Complications	Time onset	Radiological findings	Diagnostic criteria/features
Hyper-acute rejection	< 24 h	Diffuse pulmonary opacities.	It is a type of antibody-mediated rejection with no gold standard diagnostic test.
Primary graft dysfunction	0–72 h	Non-specific perihilar and basilar opacities and interstitial thickening.	Development of hypoxia and diffuse pulmonary radiographic opacities with no other identifiable cause.
Acute cellular rejection	1st week–1 year	Non-specific ground-glass opacities, consolidation, and interstitial thickening with or without pleural effusions.	Diagnosed and graded based on pathologic findings on TBB specimens; lymphohistiocytic inflammatory infiltrates centered on small blood vessels; or bronchioles.
Acute antibody-mediated rejection	1st week–1 year	No described specific imaging findings.	Presence of DSA, characteristic lung histology, and positive C4d within the graft.
Bronchiolitis obliterans Syndrome	> 1 year	Air trapping on expiratory images; bronchial wall thickening; centrilobular nodules; with or without bronchiectasis.	FEV1 decline ≥ 20% from baseline; irreversible obstructive PFT pattern. Pathology findings are characteristic but not required for diagnosis.
Restrictive allograft syndrome	> 1 year	Early: central and peripheral ground-glass opacities. Late: peripheral and upper lung predominant reticulation, architecture distortion, and traction bronchiectasis with hilar retraction.	FEV1 decline ≥ 20% from baseline; total lung capacity decline ≥ 10% from baseline; irreversible restrictive PFT pattern.

*TBB*, transbronchial biopsy; *DSA*, donor specific antigen; *C4d*, complement component 4d; *FEV*_*1*_, forced expiratory volume in 1 s; *PFT*, pulmonary function test

## Data Availability

Data sharing is not applicable to this manuscript as no datasets were generated or analyzed.
